# High Amplitude EEG Motor Potential during Repetitive Foot Movement: Possible Use and Challenges for Futuristic BCIs That Restore Mobility after Spinal Cord Injury

**DOI:** 10.3389/fnins.2017.00362

**Published:** 2017-06-23

**Authors:** Aljoscha Thomschewski, Yvonne Höller, Peter Höller, Stefan Leis, Eugen Trinka

**Affiliations:** ^1^Department of Neurology, Christian Doppler Medical Center, Paracelsus Medical UniversitySalzburg, Austria; ^2^Spinal Cord Injury and Tissue Regeneration Center SalzburgSalzburg, Austria; ^3^Department of Psychology, Paris-Lodron University of SalzburgSalzburg, Austria; ^4^Center for Cognitive Neuroscience SalzburgSalzburg, Austria

**Keywords:** spinal cord injury, neuroplasticity, brain plasticity, motor evoked potential, electroencephalography, neuroprosthetics

## Abstract

Recent advances in neuroprostheses provide us with promising ideas of how to improve the quality of life in people suffering from impaired motor functioning of upper and lower limbs. Especially for patients after spinal cord injury (SCI), futuristic devices that are controlled by thought via brain-computer interfaces (BCIs) might be of tremendous help in managing daily tasks and restoring at least some mobility. However, there are certain problems arising when trying to implement BCI technology especially in such a heterogenous patient group. A plethora of processes occurring after the injuries change the brain's structure as well as its functionality collectively referred to as neuroplasticity. These changes are very different between individuals, leading to an increasing interest to reveal the exact changes occurring after SCI. In this study we investigated event-related potentials (ERPs) derived from electroencephalography (EEG) signals recorded during the (attempted) execution and imagination of hand and foot movements in healthy subjects and patients with SCI. As ERPs and especially early components are of interest for BCI research we aimed to investigate differences between 22 healthy volunteers and 7 patients (mean age = 51.86, SD = 15.49) suffering from traumatic or non-traumatic SCI since 2–314 months (mean = 116,57, SD = 125,55). We aimed to explore differences in ERP responses as well as the general presence of component that might be of interest to further consider for incorporation into BCI research. In order to match the real-life situation of BCIs for controlling neuroprostheses, we worked on small trial numbers (<25), only. We obtained a focal potential over Pz in ten healthy participants but in none of the patients after lenient artifact rejection. The potential was characterized by a high amplitude, it correlated with the repeated movements (6 times in 6 s) and in nine subjects it significantly differed from a resting condition. Furthermore, there are strong arguments against possible confounding factors leading to the potential's appearance. This phenomenon, occurring when movements are repeatedly conducted, might represent a possible potential to be used in futuristic BCIs and further studies should try to investigate the replicability of its appearance.

## 1. Introduction

In the last years about 330,000 people in Europe suffered from spinal cord injury (SCI) with approximately 55% being tetraplegic (Ouzkỳ, 2002; Van den Berg et al., 2010). Every year up to half a million people worldwide become spinal cord injured causing a loss of independency and privacy and thus an extremely decreased quality of life (World Health Organization and International Spinal Cord Society, 2013; Rupp, 2015). Although there is a huge variety of common rehabilitative therapies as well as newer methods available for patients with SCI (e.g., Popovic et al., 2002; Nash, 2005; Harvey et al., 2016), a considerable amount of patients remain with extremely impaired motor functioning. For these patients futuristic neuroprostheses might represent a way to regain some mobility and thus increase their quality of life. Using brain-computer interfaces (BCIs) patients can learn to control prostheses or robotic devices, that are programmed to react to predefined patterns of neuronal activity, as obtainable using electroencephalography (EEG), which is the method of choice when implementing this futuristic idea (Pfurtscheller and Neuper, 2001; Pfurtscheller et al., 2006b; Hochberg et al., 2012; Jackson and Zimmermann, 2012; Collinger et al., 2013).

However, developing and implementing BCIs for patients with SCI is very challenging, especially since the loss of afferent information processing can lead to considerable changes in the brain's structure and functionality, known as neuroplasticity or brain plasticity. The general process of neuronal reorganization is an important property of the central nervous system, as it allows us to adapt to physiology, the environment and certain influences deriving from behavior (James, 1890; Karni et al., 1995; Poldrack, 2000). Furthermore, these changes may not only pertain to neurons and synapses, but also to larger networks and cortical maps (Hebb, 1949; Merzenich and Sameshima, 1993; Pascual-Leone and Torres, 1993; Elbert et al., 1995; Buonomano and Merzenich, 1998; Jain et al., 2008). Although brain plasticity is of general importance, it presents us with great problems in the case of neurological conditions such as SCI as they have great implications on the success of rehabilitative therapy and for the development of BCIs for the use in innovative neuroprostheses (Pfurtscheller et al., 2005; Conradi et al., 2009; Rupp et al., 2013).

Studies of neuroplasticity in rats show that cortical areas formerly representing a forelimb were overtaken by other motor representations such as the shoulders after a forelimb amputation (Donoghue and Sanes, 1988; Sanes et al., 1990). In human patients with SCI the sensorimotor map areas of the affected legs can be invaded by adjacent sensorimotor maps (Bruehlmeier et al., 1998; Perani et al., 2001) and motor areas can deteriorate (Wrigley et al., 2009). Furthermore, findings suggest that plastic changes happen rapidly within minutes (Brasil-Neto et al., 1993; Aguilar et al., 2010), and depend on the degree of deafferentation (Rossi et al., 1998). Given the numerous possible changes occurring after SCI, a better understanding of these processes is very desirable in order to improve therapeutic treatment and to develop reliable brain computer interfaces (Ziemann et al., 1998; Ding et al., 2005; Dietz, 2012).

Another problem of futuristic BCIs so far is that they suffer from slow response times, thus it was suggested to investigate brain activity related to early movement initiation and execution, such as motor potentials (Krauledat et al., 2004). So far two components of motor related potentials obtained using EEG occurring previous to a voluntary movement have been widely reported (Cunnington et al., 1995; Cui et al., 1999). An early component known as the readiness potential (Kornhuber and Deecke, 1965, 2016) is characterized by a negative potential slowly evolving in the 2 s preceding a movement. This component is bilaterally represented and predominantly appears over the vertex electrode Cz, thus being associated with activity of the supplementary motor area (Shibasaki et al., 1980; Tamas and Shibasaki, 1985). A second component, considered to be triggered by the first one (Deecke, 1987), contains a rapid peak in negative amplitude measured about 500 ms before movement onset with a maximum over contralateral motor regions associated with activated muscle groups (Boschert et al., 1983). With respect to SCI and plastic changes, the motor potentials seem to be shifting with the peak especially found more posterior in patients with SCI (Green et al., 1998, 2003; Castro et al., 2007; Gourab and Schmit, 2010). The posterior shift was also found to be reversed in the course of recovery and to have “a significant relationship to prognosis in paraplegia” (Green et al., 1999). Despite their obvious benefit for BCI application when applied successfully as shown recently (Jeong et al., 2017), motor related potentials are often associated with high trial numbers, since they often require a training period (e.g., Kornhuber and Deecke, 1965, 2016).

In summary, the motor related EEG potentials seem an interesting marker both to further explore plastic changes occurring after SCI and also to consider for implementation in futuristic BCIs, aiming to restore mobility in patients with SCI. Therefore, we analyzed data obtained in a previous study investigating plastic changes in general, to gain an exploratory view on possible ERP potentials that might be of interest for further studies with respect to BCI implementation. Finding an interesting oscillation elicited during foot movement execution we focused on exploring this potential and tried to assess whether it could be obtained on a small number of trials, since this obviously would be desirable for the use in neuroprosthetics.

## 2. Methods

### 2.1. Subjects

Twenty-two healthy volunteers and seven patients with traumatic SCI resulting in paraplegia, tetraplegia or tetraparesis took part in a study at the Department of Neurology in the Christian Doppler Medical Centre Salzburg. The healthy subjects were aged from 19 to 35 years (mean = 23.14, SD = 3.4) and 14 of them were women (64%). Healthy volunteers were recruited by e-mail amongst students of the universities of Salzburg. The patient group only consisted of male subjects, aged between 24 and 70 years (mean = 51.86, SD = 15.49). Patients with cervical and thoracic lesions were included regardless of the duration of their SCI. All patients were right-handed according to their anamnesis interviews. Details about the patients participating in this study can be found in Table 1. The study was approved by the local ethics committee (Ethics Commission Salzburg; number E-Nr1541) and was conducted in accordance with the guidelines proposed in the Declaration of Helsinki. Written informed consent was obtained from all participants.

**Table 1:** Patients participating in the study.

**Subject**	**Age (years)**	**ASIA score**	**Level of Injury**	**Duration of injury (months)**	**Mobility status**
Patient 1	51	D	C4	2	Wheelchair
Patient 2	61	C	C4	204	Wheelchair
Patient 3	24	C	C5	48	Wheelchair
Patient 4	65	C	C4	19	Power wheelchair
Patient 5	44	D	C6	216	Wheelchair
Patient 6	48	D	C7	314	Wheelchair
Patient 7	70	D	T8	13	Wheelchair

### 2.2. Experimental procedure

After completing questionnaires about their demographic data and their ability to imagine certain movements participants took part in an EEG experiment. During the EEG recordings participants were asked to observe a movement, imagine performing a movement and to actually execute or attempt to execute a movement. Two movements were used in this experiment: tapping the right foot and clenching the right hand. Additionally, there was a resting condition where participants were asked not to move at all.

**Figure 1:**
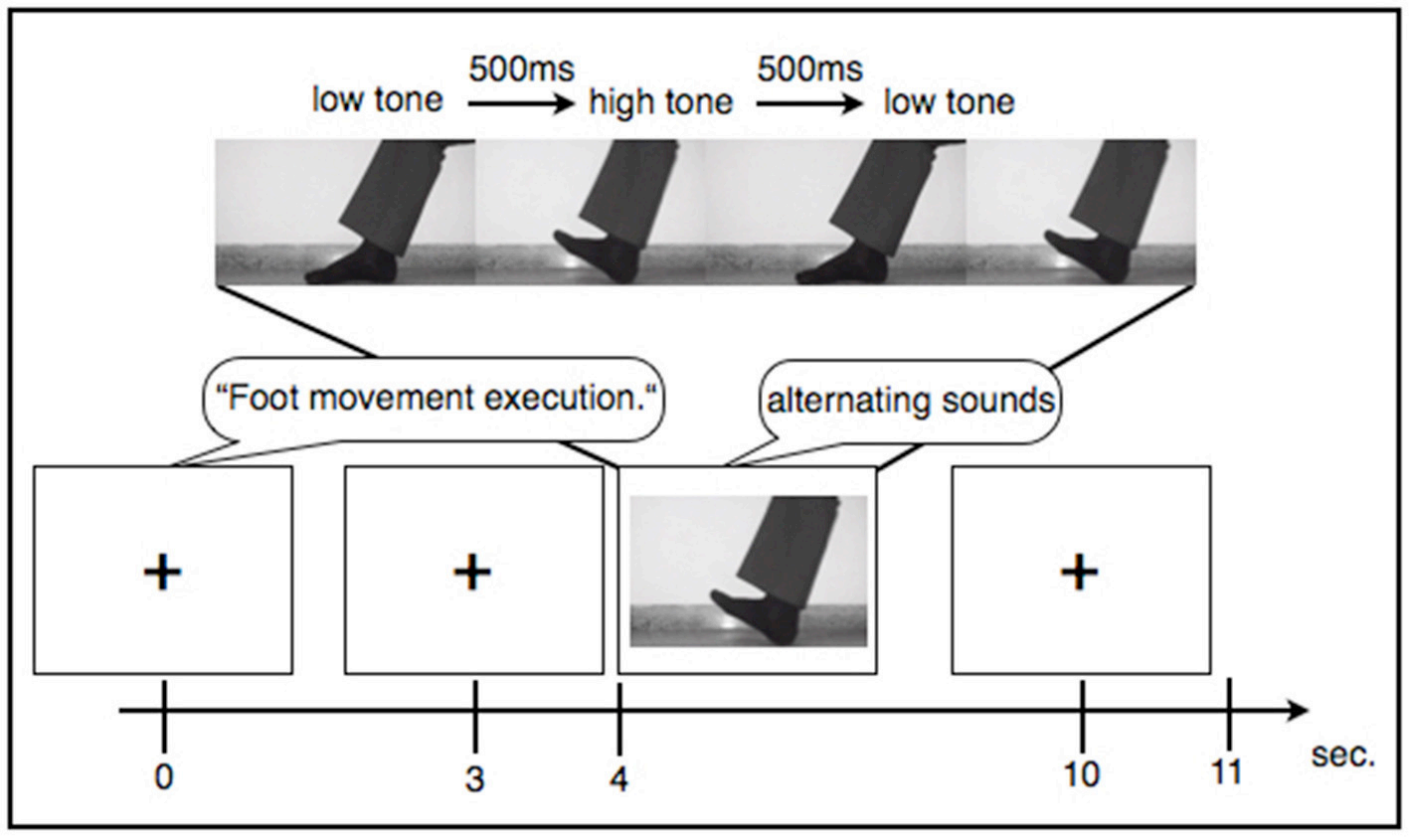
Example sequence of an experimental trial.

During each condition participants saw a fixation cross on a blank screen, except during the observation condition where they saw somebody performing the movements. During all experimental conditions the participants were asked to perform the tasks according to a rhythm of two alternating sounds. Also during resting, participants heard the two short “beep” sounds with different pitch levels alternating at a frequency of 1 Hz. The alternating sounds were displayed for 6 s, thus suggesting six consecutive movements at a frequency of 1 Hz. The paradigm contained 25 trials for each condition that were displayed in a random order. Each trial consisted of audio instructions that lasted 3 s (e.g., “hand movement: execution”) followed by a pause of 1 s. After the pause the experimental condition started indicated by a startle sound. In the following 6 s a low pitched tone indicated to lift the forefoot or to clench the hand and a higher pitched tone displayed 500 me later indicated to lower the foot or to loosen the fist again (see Figure 1). Trials were again separated by a general pause of 1 s. Prior to the experiment all participants listened to a standardized general instruction and saw an example trial for each condition. A whole experimental session including the general instructions lasted about 39 min. Subjects were comfortably seated at a distance of 50 cm from an 11-inch monitor displaying the experiment. Sounds were emitted directly from the computer at the same intensity for every participant. Patients 1, 2, 3, and 4 they took part in an additional study investigating the effects of regular transcranial magnetic stimulation over M1 on EEG connectivity markers as well as on psychomotor functions. Due to this, these four patients had multiple recordings using the same experimental paradigm all of which were included in the analysis.

### 2.3. Data acquisition and general preprocessing

**Figure 2:**
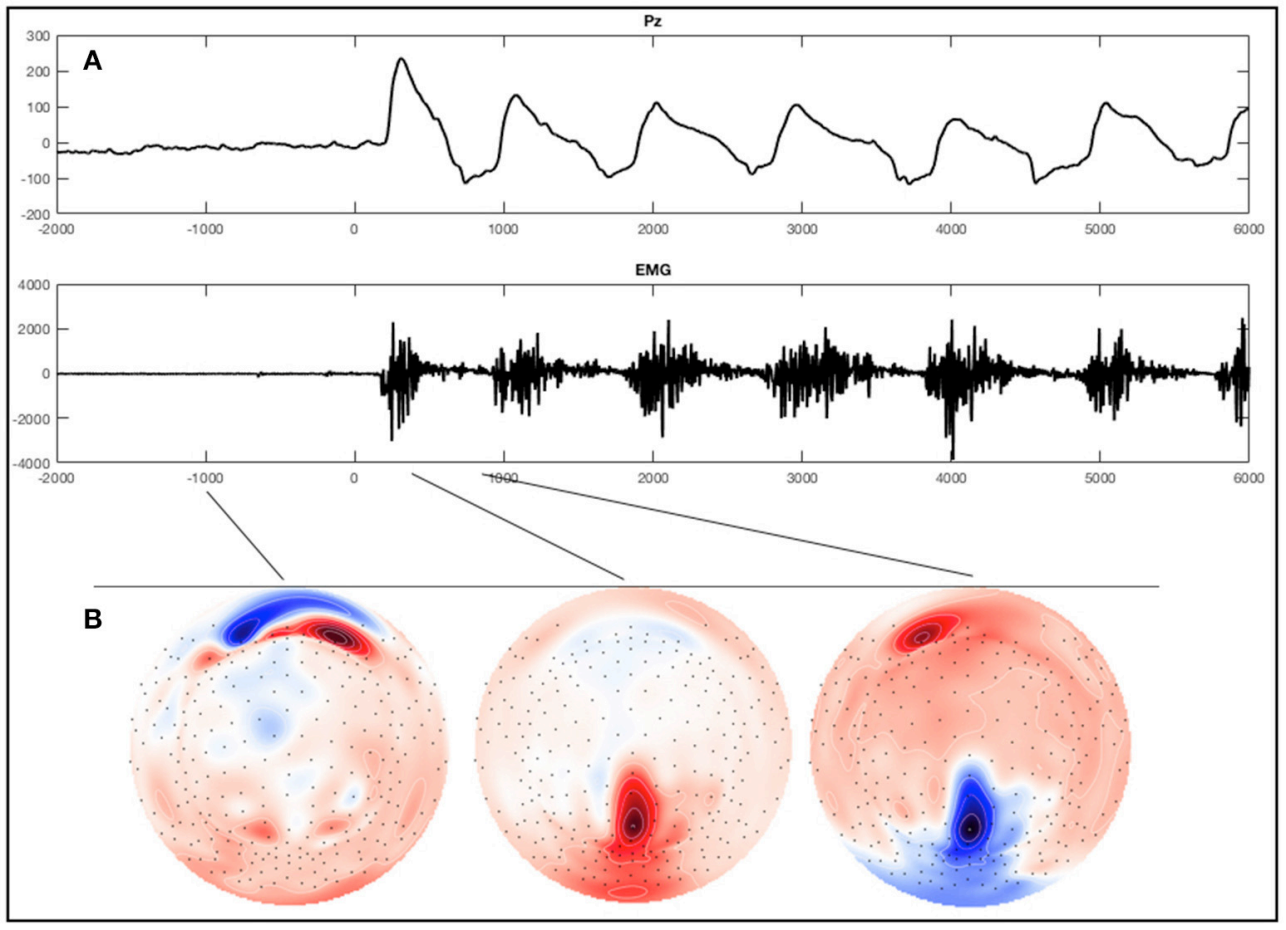
Example of an ERP of a healthy subject during the foot movement condition. On the top **(A)** a single trial response on Pz and the EMG with regard to stimulus onset (time point 0) is shown. The x-axes display time (ms) and the y-axes amplitudes (μV). On the bottom **(B)**, activation maps of the averaged signal can be seen.

EEG was recorded with 256-channel HydroCel geodesic sensor nets and a GES 300 amplifier (Electrical Geodesic Inc., EGI, Eugene, OR). Electrode Cz served as reference. Data was recorded at 250 Hz sampling rate using EGI's NetStation 4.5.6 software. With respect to the instructions provided by EGI and taking the input impedance of the amplifier into account (Ferree et al., 2001; Geodesics, 2007), we kept impedances below 75 kΩ, in accordance with the current guidelines proposed by the Society for Psychophysiological Research (Keil et al., 2014). In addition to the scalp electrodes we used a polygraphic input box to directly record an electrocardiography and an electromyography (EMG) using two Ag/AgCl electrodes each. For the EMG the electrodes were consequently placed over the musculus tibialis anterior on the right leg for all participants. The differential signal of these additional electrode paris were recorded as two additional channels alongside the EEG. Data analysis for EEG channels was conducted in accordance with other studies examining ERPs, using EGI's NetStation software as well as the NetStation user manual provided by EGI (Leppänen et al., 2007; Casanova et al., 2012; Espinet et al., 2012). After administration of a 0.1 Hz highpass IIR filter and a 30 Hz FIR lowpass filter the data was segmented according to the five conditions: foot tapping execution, foot tapping imagination, hand clenching execution, hand clenching imagination, and resting. The movement observation conditions were excluded from this analysis. For data segmentation we used the beginning of the sound stimuli indicating the first movement to be imagined or executed. When an EMG signal was present (that is, during foot movement execution), segments were defined according to the beginning of muscle activity (see Figure 2A for an example). Markers were then set manually after visually detecting the first muscle jerk in the foot movement condition. In three healthy subjects one to three segments of the foot movement condition were excluded since no sufficient muscle activity was detectable. Segments started 2 s before the sound stimuli or muscle activity and lasted for 8 s. After segmentation the all segments were baseline corrected using the first 2 s as a baseline.

### 2.4. Artifact rejection

EEG channels were automatically marked bad if the fast average amplitude exceeded 200μV within a moving window of 100 ms. Following bad channel detection, eye blinks and eye movements above a threshold of 100μV were also marked automatically using the vertical and horizontal eye channels that are contained in the sensor nets. We conducted two data analyses since a strict exclusion of segments would have led to a profound loss of data. In a strict artifact exclusion we rejected all segments that (i) contained an eye blink, (ii) contained an eye movement, or (iii) in which the channel Pz was marked bad. This is further referred to as strict exclusion. In a second analysis we only excluded segments in which Pz was marked bad, which is further referred to as lenient exclusion. The amount of included segments for each condition and subject are can be seen in Table 2 for strict artifact exclusion and in Table 3 for lenient artifact exclusion.

**Table 2:** Number of included segments per condition after strict artifact exclusion (number of recorded segments if not 25).

**Subject**	BEWG^*^	MFOT^*^	MHND^*^	IFOT^*^	IHND^*^	REST^*^
Patient 1		4 (50)	7 (50)	13 (50)	13 (50)	8 (50)
Patient 2		0 (100)	0 (100)	0 (100)	0 (100)	0 (100)
Patient 3		0 (64)	0 (64)	0 (64)	0 (62)	0 (64)
Patient 4		20 (75)	3 (75)	22 (75)	22 (75)	17 (75)
Patient 5		2	0	0	2	1
Patient 6		0	0	0	0	0
Patient 7	0 (24)	1	0	0	0	1
Control 1	1	1	0	0	0	0
Control 2	0	2	4	7	10	7
Control 3	0	0	0	1	0	2
Control 4	0 (23)	0	0	2	1	3
Control 5	7	7	9	11	15	11
Control 6	9	9	11	10	14	5
Control 7	0	0	1	0	4	0
Control 8	3	3	4	4	7	0
Control 9	8	8	5	14	11	1
Control 10	0	0	0	0	0	3
Control 11	0	0	0	0	0	1
Control 12	0	0	0	0	0	0
Control 13	0 (24)	0	0	0	0	0
Control 14	1	0	0	1	1	2
Control 15	9	9	13	17	19	13
Control 16	1	1	0	0	1	0
Control 17	0	0	0	1	0	1
Control 18	8	8	8	10	12	7
Control 19	1	1	0	5	8	9
Control 20	0	0	0	0	0	0
Control 21	0 (22)	0	0	0	0	0
Control 22	5	6	7	13	15	17

* *BEWG, foot movement locked to EMG; MFOT, foot movement locked to stimulus onset; MHND, hand movement; IFOT, foot movement imagination; IHND, hand movement imagination; REST, resting. Subjects with no included segments are not depicted*.

**Table 3:** Number of included segments per condition after lenient artifact exclusion (number of recorded segments if not 25).

**Subject**	BEWG^*^	MFOT^*^	MHND^*^	IFOT^*^	IHND^*^	REST^*^
Patient 1		50 (50)	50 (50)	50 (50)	50 (50)	50 (50)
Patient 2		99 (100)	100 (100)	100 (100)	98 (100)	98 (100)
Patient 3		63 (64)	63 (64)	59 (64)	61 (62)	57 (64)
Patient 4		75 (75)	75 (75)	75 (75)	75 (75)	75 (75)
Patient 5		25	25	25	25	25
Patient 6		25	25	25	25	25
Patient 7	24 (24)	25	25	25	25	25
Control 1	24	24	25	25	25	24
Control 2	25	25	25	25	25	25
Control 3	25	25	25	25	25	25
Control 4	23 (23)	25	25	25	25	25
Control 5	25	25	25	25	25	25
Control 6	25	25	25	25	25	25
Control 7	25	25	25	25	25	25
Control 8	25	25	25	25	25	25
Control 9	25	25	25	25	25	25
Control 10	17	17	25	25	25	25
Control 11	22	22	22	22	24	22
Control 12	25	25	25	25	25	25
Control 13	24 (24)	25	25	25	25	25
Control 14	24	24	25	25	25	25
Control 15	25	25	25	25	25	25
Control 16	24	24	25	25	24	23
Control 17	25	25	24	25	25	25
Control 18	25	25	25	25	25	25
Control 19	24	24	25	25	25	25
Control 20	25	25	25	25	25	25
Control 21	22 (22)	25	25	25	25	25
Control 22	25	25	24	25	25	25

* *BEWG, foot movement locked to EMG; MFOT, foot movement locked to stimulus onset; MHND, hand movement; IFOT, foot movement imagination; IHND, hand movement imagination; REST, resting*.

### 2.5. EEG data analysis

For visual inspection the data was re-referenced against both mastoids. Segments were averaged for each condition across trials, individually for each subject. Screening all 256 electrodes in the averages of healthy subjects and patients with SCI in all conditions we could not detect a distinct component resembling motor related potentials described in the literature. We did find a hint of a negative component in some healthy subjects and in two patients at the beginning of the conditions around the electrodes F3 and F4. However, this component was very inconsistent and the peak only had an amplitude of about only -4μV. Interestingly though we did see a striking oscillation in the parieto-central region (Figure 2B), which was maximal over the electrode Pz during the execution of foot movements in most healthy subjects. Thus, we focused further investigations on this electrode and conducted a statistical analysis examining the differences in ERP signals obtained from this electrode during the experimental conditions compared to rest. Pz refers to a montage equivalent to the international 10-10-electrode position as described by Luu and Ferree (2005).

For statistical analysis of the EEG data we used a common average montage. Since single trials are relevant for BCIs, we focused on the comparison of single trials and conducted permutation tests comparing the ERPs elicited on Pz of all trials for each experimental condition with the resting condition on a single-subject level (Maris and Oostenveld, 2007). The critical alpha-level was set to 0.05 and the Monte Carlo *p*-values were calculated on 2000 random partitions using Matlab® (Version R2010b, The Mathworks). Before statistical calculations all segments were mean-corrected within Matlab®. The critical thresholds for differences to be considered significant after both strict and lenient artifact rejection are provided in the supplementary section.

## 3. Results

### 3.1. Analysis after strict artifact exclusion

#### 3.1.1. Healthy subjects

**Figure 3:**
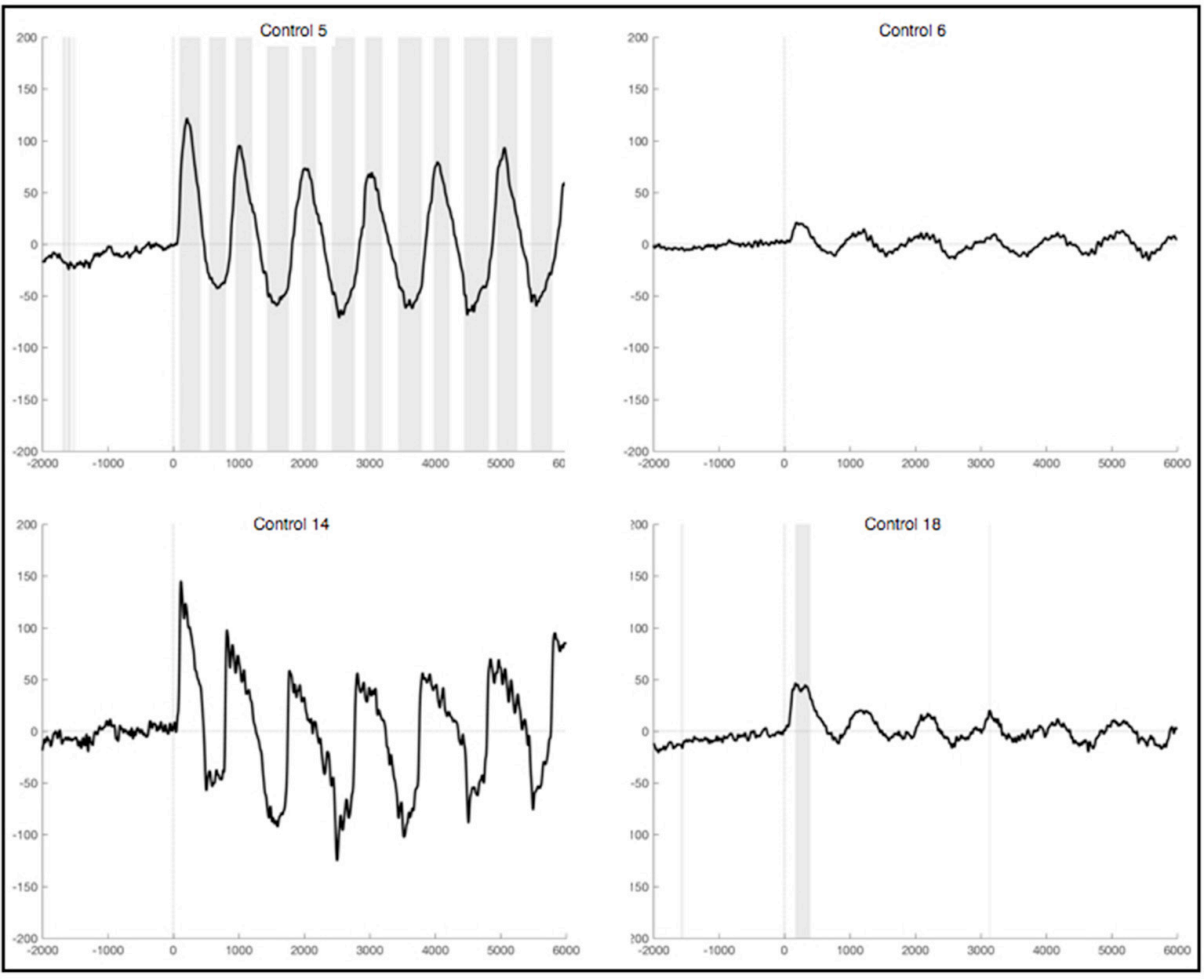
Average ERPs of four healthy subjects recorded on Pz during foot movement execution. Segments are locked to EMG onset (time point 0) and artifacts had been excluded strictly. The x-axis displays time (ms) and the y-axis amplitudes (μV). Sections shaded in gray indicate significant differences from the resting condition on a single trial level with *p* <0.05.

**Figure 4:**
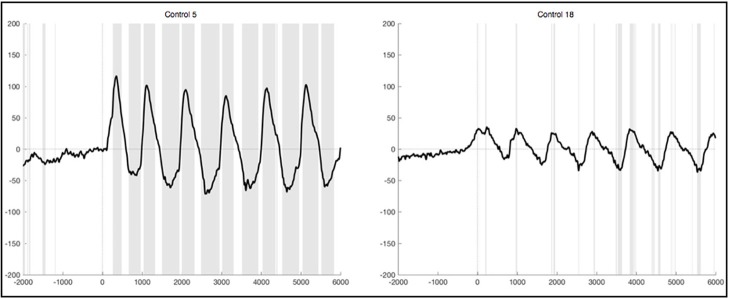
Average ERPs of two healthy subjects recorded on Pz during foot movement execution. Segments are based on stimulus onset (time point 0) and artifacts were excluded strictly. The x-axis displays time (ms) and the y-axis amplitudes (μV). Sections shaded in gray indicate significant differences from the resting condition on a single trial level with *p* < 0.05.

The observed oscillation during foot movement execution was characterized by high positive amplitudes and had a frequency of about 1 Hz. When looking at the segments locked to the EMG signal, we found the oscillation to be time-locked to the rhythm of the leg movements (Figure 3). Furthermore, the oscillation lasted 6 s, which resembles the duration of the movement condition. After strict artifact exclusion we obtained this oscillation in five out of 11 healthy subjects, who provided artifact free segments. In two of these five subjects, all oscillations during the 6 s differed significantly from the resting condition. In another two subjects only the first sinusoidal oscillations reached statistical significance. In general, the oscillation was most distinct at the first movements of the participants and got weaker in amplitude throughout the six movements of one trial. Also, the amplitudes differed between the trials within one subject as well as between subjects. When observing the ERPs calculated by using the segments based on stimulus onset and not on EMG activity, the pattern was still visible in four subjects, though not that distinct and with smaller amplitudes (Figure 4). And only in two of these subjects did the peaks reach statistical significance in comparison to the resting condition segments.

During the hand movement condition, there were no distinct potentials visible after strict exclusion of contaminated segment and only ten subjects had artifact free segments suitable for analysis. During the imagination of foot and hand movements there also was no distinct oscillatory pattern detectable, despite the suggestion of similar activation during imagination and execution of movements in the literature (Beisteiner et al., 1995; Decety, 1996; Pfurtscheller and Neuper, 1997; Lotze et al., 1999; Pineda et al., 2000). With the exception of one subject, statistical significance testing did not reveal any differences between the ERPs elicited during resting and during the imagination conditions. In that one subject two sections of the ERP reached statistical significant differences to the resting condition, but no distinct components were visible.

#### 3.1.2. Patient group

**Figure 5:**
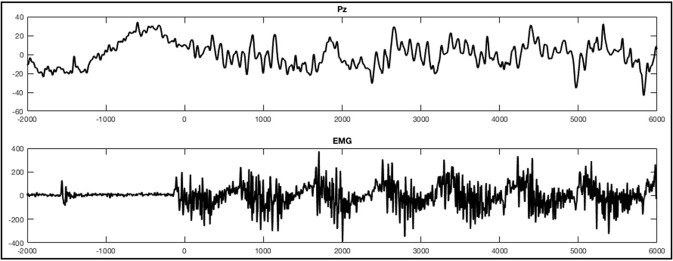
ERPs of a single trial during the foot movement condition of patient 7. The upper figure depicts the response on Pz with regard to stimulus onset (time point 0) and the lower figure shows the EMG responses. The x-axes display time (ms) and the y-axes amplitudes (μV).

Patients 1, 2, 3, 5, and 6 yielded EMG signals with fast but low amplitudic activity considered to be noise. There were no changes in the EMG signal during experimental conditions in comparison to rest and no rhythmic bursts during the foot movement condition. In between there were singly bursts visible but they had no relation in time to any stimuli or experimental condition and were thus considered to reflect occasional spastic jerks. Patient 4 yielded nearly no activity at all and his EMG signal was characterized by very flat lines suggesting no muscle activity at all. Patient 7, however, elicited a rhythmic bursting during the foot movement condition that resembled the frequency of the two alternating sound that were displayed (Figure 5).

With regard to the EEG patients with SCI seemed to generally produce a greater amount of artifacts, especially eye movements during the two execution conditions, leading to three patients having all segments removed during strict artifact rejection. Thus only four patients remained and in these only a few segments could be analyzed. Accordingly we did not obtain any specific ERPs from the EEG recorded during the four experimental conditions.

### 3.2. Analysis after lenient artifact exclusion

#### 3.2.1. Healthy subjects

**Figure 6:**
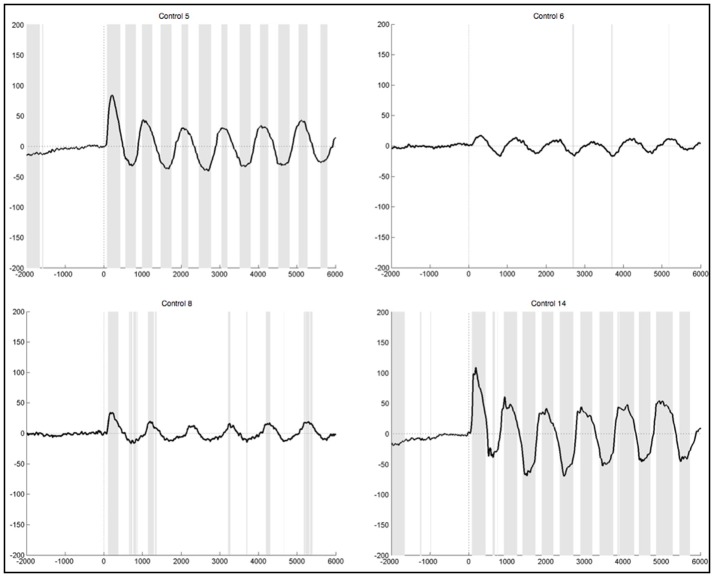
Average ERPs of four healthy subjects after lenient exclusion recorded on Pz during foot movement execution. Segments are based on EMG onset (time point 0). The x-axis displays time (ms) and the y-axis amplitudes (μV). Sections shaded in gray indicate significant differences from the resting condition on a single trial level with *p* < 0.05.

After lenient exclusion, there was a 1 Hz oscillations visible in ten healthy subjects when segments were EMG-locked (Figure 6). In four of these subjects, all oscillations during the 6 s differed significantly from the resting condition. In two subjects not all six peaks reached significance, in three subjects only the first peaks, and one subject yielded no significant difference in the ERPs elicited during foot movement compared to rest. Another subject yielded traces of a 1 Hz oscillation, though not distinct and also not significantly different from resting. In general, the characteristics remained the same with high positive amplitudes elicited time locked to the first sound indicating to lift the foot and a rhythmic activity in line with the six movements, though the amplitudes were quite higher when leniently rejecting segments. When segments were based on stimulus onset the initial positive peak was even higher in some subjects, once reaching up to 200μV in the averaged ERPs of one healthy control. Despite the higher amplitudes and accordingly more significantly different peaks there were no further differences between the results from segments that were EMG-locked and segments based on stimulus onset.

After leniently excluding segments that were contaminated with artifacts, we still could not observe specific potentials or oscillatory patterns in neither of the remaining three conditions, hand movement execution, as well as imagination of hand or foot movements.

#### 3.2.2. Patient group

**Figure 7:**
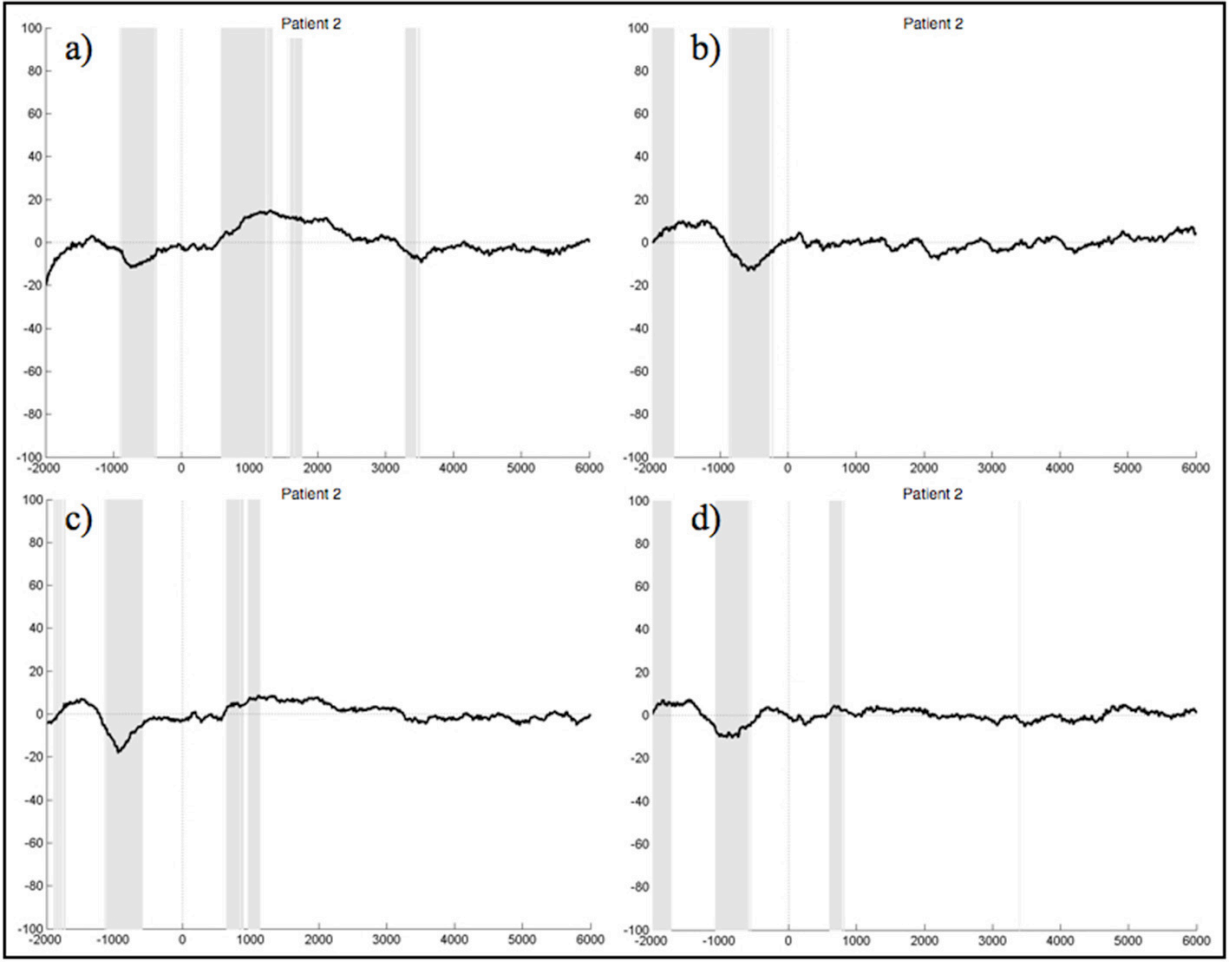
Average ERPs of patient 2 recorded on the electrode Pz during the foot **(a)** and hand **(c)** movement execution conditions and during the imagination of foot **(b)** and hand **(d)** movements. Conditions start at time 0. Segments are not corrected for artifacts. The x-axis displays time (ms) and the y-axis amplitudes (μV). Gray shading indicates significant differences from the resting condition on a single trial level with *p* < 0.05.

When segments containing bulbar artifacts were not rejected, patient 1 still yielded no clear ERPs during the execution and imagination of either foot and hand movements, although the upper extremities were unimpaired. In the ERPs of patient 2 there was a negative component elicited about 1 s prior to stimulus start during all four experimental conditions, which also significantly differed from the resting condition (Figure 7). The ERPs of patients 3, 5, and 6 did not reveal any distinct components at all. There were also no sections reaching statistical significance compared to rest. In the ERPs recorded in patient 4 we could not detect any distinct pattern during the four experimental conditions, other than significant differences to the signals obtained during rest, starting around 3.5 s after stimulus onset. Although we locked the EEG signal during the foot movement condition to the EMG response, no 1 Hz pattern was detectable in the ERPs of patient 7 during foot movement. In all four conditions no significant differences to the resting conditions could be detected.

## 4. Discussion

The study aimed to explore event-related motor responses in relation to movement execution and imagination in a patients with SCI and healthy subjects. Having a restricted set of data it was of special interest whether a distinct motor potential could be obtained using a small number of trials only. Further, a strict as well as a lenient procedure of artifact rejection should establish whether any potential found, could also be obtained without extensive preprocessing, and thus be of interest for further considerations with regard to an implementation into futuristic BCI technology.

We did find an interesting oscillation of 1 Hz during the foot movement condition in most healthy subjects over Pz. The oscillation's frequency resembled the given cadence the participants were asked to follow while performing the tasks. In comparison to the readiness potential described in the literature this oscillation is similar to the evoked signal found on posterior electrodes during repetitive leg movements (Kornhuber and Deecke, 1965). Given the frequency and the exact time range, which this pattern can be observed in, this component can be considered to reflect a motor-related potential. Additionally, we can exclude that it is elicited due to auditory processing of the acoustic stimuli or general external artifacts, since it is not present during the resting condition, in which participants also listened to the auditory stimuli. However, the oscillatory pattern consists of positive and not negative peaks in most subjects, whereas literature would suggest negative components being predominant (Kornhuber and Deecke, 1965). Only in three subjects the component started with a negative peak and the amplitudes of the positive phases were generally higher than the negative ones. Thus, we were not able to obtain the same motor potential as described in previous studies (Shibasaki et al., 1980; Boschert et al., 1983; Tamas and Shibasaki, 1985; Deecke, 1987). Furthermore, we were not able to obtain this oscillation in patients with SCI. Still, in many healthy subjects the oscillation was obtainable using only a small number of trials and it was very distinct even with lenient artifact rejection procedures, making it a possible candidate for a use in BCIs. Nonetheless, some questions come to mind given our results: First, why did we not find potentials similar to those reported in the literature? Second, could the high amplitudes obtained reflect a physiological signal or are they merely artificial? Third, why did we obtain this pattern only during foot movement execution? And fourth, why did we not obtain this component in any of the patients with SCI?

The answer as to why we did not find potentials similar to those reported in the literature may lie in the small trial number of this study. Taking into consideration that existing studies obtained motor potentials by averaging more than 100 trials, and even up to 500 in the first study on the readiness potential by Kornhuber and Deecke (1965), it is reasonable that with only 25 trials we were not able to detect the same ERP components. Other studies reported an attenuation of motor potentials in the course of experiments (Freude and Ullsperger, 1987; Dirnberger et al., 2004), which is interesting, since our findings suggest that the positive amplitudes we detected decreased in the course of the six movements. Keeping in mind, that the oscillation we found was not present in all trials, even in the subjects eliciting the highest amplitudes, a high number of trials containing a negative readiness potential, might have led to the oscillation being averaged out, if the number of trials showing positive amplitudes decreased in relation. Furthermore, a habituation effect might also cause the decrease in positive amplitude over repeated movements. However, this is pure speculation at this point and needs to be investigated in future studies examining this component in a larger number of trials, to determine the number of trials necessary to robustly evoke this component.

Concerning the cause of such high amplitudes, it can be agreed on that the high amplitudes we obtained seem artificially high at first glance. However, there are several components of the EEG known to have high amplitudes, which can also be seen on single trials and are definitely based on physiological mechanisms: like K-Complexes often exceeding amplitudes of 200μV (Colrain, 2005), hypsarrhytmia (>300μV), three per second spike-and-slow-wave complexes (up to 1,000μV), or vertex sharp transients (up to 250μV; Noachtar et al., 1999). Given these other phenomena eliciting high amplitudes, we cannot discard the obtained component purely on the basis of high amplitudes. Furthermore, we can rule out other confounding factors like the contamination with artifacts (Tatum et al., 2008; Hamer et al., 2010). Eye-blinks and eye-movement artifacts as a cause of the appearance can be ruled out, since the potential was well visible and statistically significant in some cases, even when they were controlled for in a very strict manner. Electrode artifacts would have appeared in other conditions as well and are characterized by different attributes. The same can be said about external artifacts, created by electrical devices for instance. Ruling out artificial EEG responses and also accepting the fact that it is very unlikely that half of our healthy subjects suffered from an undiagnosed condition leading to pathological EEG phenomena, one could hypothesize that the obtained potential is a normal physiological response to repeated movements of the lower limb. Unfortunately, we do not know of any description of such high amplitudes in connection with motor-related EEG responses.

The question why we were only able to obtain this distinct oscillation in response to lower limb movement execution can not be fully answered and needs further studies on that matter. However, due to the cortical representations of the lower limbs, it makes sense that foot movements result in motor potentials with higher amplitudes when measured over central electrodes compared to hand movements (Penfield et al., 1954; Luft et al., 2002; Lippert, 2006). The different locations of the cortical representations in the sensory and motor cortices might explain why we detected the 1 Hz oscillations more clearly on centro-parietal electrodes during foot movement conditions than during hand movements, but it does not explain why we did not observe it during the hand movement execution on other electrodes. The fact, that we did not find a similar potential during the hand movement condition, is a strong argument that the obtained potential might be an artifact after all. The leg movement might elicit an artifact by shaking the chair in which healthy participants sat. This would also explain, why patients with SCI did not elicit this component. However, such an artifact would probably be visible on a larger scale and not only on Pz and surrounding electrodes. Interestingly, we did not find this distinct oscillatory pattern during the imagination of foot movements either. This might be explained by the facts that motor potentials during movement imagination have lower amplitudes in general (Beisteiner et al., 1995), and that the ability to perform motor imagery highly varies across subjects (Pfurtscheller et al., 2006a). We also did not find significant sections in all participants yielding the 1 Hz-oscillation in the experimental conditions' ERPs compared to the resting condition, but as stated by Maris and Oostenveld (2007), permutation significance testing is a very conservative method in the sense that it is not as sensitive as the “uncorrected *p*-value approach.”

Finally, the reason why we did not obtain the oscillation in patients with SCI might be caused by neuroplasticity. Especially since all patients, who participated in this study, suffered from impaired motor and sensory functions of the lower limbs. Thus, at least a decreased response was expected. However, such a great difference between healthy controls and patients could not have been anticipated, taking into account that for example patient 1 was tested only 2 months after his injury. Considering that plastic changes depend on factors like the level of injury, time since injury, the extent of recovery or even experimental manipulations (Raineteau and Schwab, 2001; Kokotilo et al., 2009), additional investigations of this potential with alternative paradigms are needed to further explain the absence of the potential in our patient sample. For example, a patient group with more incomplete paresis and thus a greater amount of preserved motor functions would of interest to further investigate on that matter. Also the execution or an attempt of larger leg movements might be a reasonable next step.

Having a small and heterogenous group of patients with very different patients is also the first limitation of our study. Furthermore, our healthy subjects greatly differed from the patient group with regard to age and gender. This might have fostered the discrepancy in results between the group. Second, we only recorded a small number of trials, and while this might be of interest when investigating the suitability of ERP markers for future BCI implementation, a larger number of trials would have been helpful to purely investigate the obtained oscillation. Also the trial number was picked arbitrarily as the paradigm for this study was not solemnly implemented for ERP analyses. Third, we had a lot of trials contaminated with artifacts, most of all eye movements and eye blinks. Although we asked participants to fixate a cross on the screen, which as suggested by the literature greatly reduces eye artifacts (Aeschbach et al., 1997; Picton et al., 2000), especially the patients' number of trials was highly reduced due to artifact rejection. On the background of examining EEG markers for a possible implementation in futuristic BCIs, artifacts will be a problem that has to be accounted for in the future and will be one of the main challenges for further research. Fourth, we only recorded the EMG on the right leg and did not place it individually for every patient. This lead to a loss of segments with usable EMG signals and thus a smaller amount of segments was eligible for EMG-locked analysis.

## 5. Conclusion

To summarize our results on the background of the proposed aims of this study, we found a distinct oscillation during the repeated execution of foot movements, obtainable using only a small number of trials. Furthermore, even with a very lenient artifact rejection and very little pre-processing, the potential was observable. Thus it seems rather robust against artifacts. Purely on the basis of these characteristics, the potential might be of interest to the community with regard to implementation in futuristic BCI-controlled neuroprostheses. However, not all healthy participants elicited this potential and none of our patients with SCI did. It is thus necessary to further investigate this movement-related potential and the replicability of our findings. Differences in brain activity between subjects, that possibly arise from neuroplastic changes due to SCI are furthermore the main problem when investigating EEG responses in this patient group and will provide us with a major challenge when trying to develop innovative neuroprostheses in the future.

## Author contributions

AT conducted the EEG recordings of the study, analyzed the data and wrote the manuscript for submission. YH provided support with data analysis and planned the project together with PH and AT. PH supported the EEG recordings and provided technical help in the setup of the experiment. SL and ET recruited patients for the study and provided medical guidance for the experiment as well as the drafting of the manuscript. All authors read and helped to improve the manuscript.

### Conflict of interest statement

The authors declare that the research was conducted in the absence of any commercial or financial relationships that could be construed as a potential conflict of interest.
